# Sit-to-Stand Navicular Drop Test-Based Injury Risk Zones Derived from a U-Shaped Relationship in Male University Athletes

**DOI:** 10.3390/jcm15031027

**Published:** 2026-01-27

**Authors:** Jarosław Domaradzki

**Affiliations:** Department of Biological Principles of Physical Activity, Wroclaw University of Health and Sport Sciences, 51-612 Wrocław, Poland; jaroslaw.domaradzki@awf.wroc.pl

**Keywords:** functional assessment, sit-to-stand navicular drop test, predictive analytics, musculoskeletal injuries

## Abstract

**Background/Objectives**: Foot mobility is considered an intrinsic risk factor for lower-limb injury, yet commonly used pronated/neutral/supinated classifications rely on arbitrary cut-points. This study aimed to develop a data-driven framework for characterizing a continuous SSNDT–injury risk gradient and deriving clinically interpretable relative-risk bands that define practical injury risk zones along the sit-to-stand navicular drop test (SSNDT) continuum. **Methods**: Data from 137 physically active male students (274 feet) were analyzed. Intra-rater reliability of the sit-to-stand navicular drop test (SSNDT) was assessed using ICC(3,1). A quadratic mixed-effects logistic regression model was used to characterize the SSNDT–injury relationship and derive odds-ratio-based risk bands for interpretive and screening purposes. **Results**: SSNDT demonstrated good intra-rater reliability (ICC(3,1) = 0.82). Model comparison supported a non-linear, U-shaped association between SSNDT and injury risk, with a minimum risk value at approximately 5.5 mm. Bootstrap analysis supported a smooth continuous risk gradient. Four representative OR levels (1.2, 1.5, 1.8, and 2.0) were selected to define SSNDT-based interpretative risk bands. Injury prevalence showed an overall increasing trend across these zones, ranging from 4.2% in the Safe zone to 52.4% in the Extreme zone. **Conclusions**: SSNDT provides a robust, data-driven basis for quantifying foot-mobility–related injury risk along a continuous non-linear gradient and for deriving clinically interpretable relative-risk bands grounded in a validated model. The proposed framework avoids arbitrary cut-points and supports individualized risk screening.

## 1. Introduction

Musculoskeletal injuries remain a major barrier to maintaining continuous physical activity among recreationally active individuals, including university students and amateur athletes. A quarter of participants report an injury with most being activity-related, affecting lower extremities like the knee, and leading to cessation of exercise in some cases [1]. In consequence, musculoskeletal injuries, particularly related to lower limb, inhibit physical activity [2]. Overuse injuries involve repetitive overload, causing damage and pain to various joints, affecting performance and ability to continue participation [3]. Many authors have focused their interests on foot mobility—particularly the functional deformation of the medial longitudinal arch—mainly due to its role in load absorption, force transfer, and neuromuscular control [4,5,6,7].

Existing models of injury risk typically assume linear effects between candidate predictors and outcomes [8]. Nonlinear spline-based analyses suggest that linear models may underestimate injury risk when predictors exhibit nonmonotonic effects [9]. However, many biomechanical and physiological determinants influence risk in a non-linear, often curvilinear fashion, where both insufficient and excessive values may be detrimental [10]. Despite this, most studies evaluating foot mobility or arch deformation still rely on binary cut-offs or linear regression models, overlooking the possibility that injury risk may vary continuously and asymmetrically across the full SSNDT continuum [11,12,13].

The sit-to-stand navicular drop test (SSNDT), as a modification of classic navicular drop test (NDT) is a widely used measure of functional foot mobility, capturing the displacement of the navicular bone between subtalar neutral and weight-bearing positions [14,15,16]. Previous studies have reported associations between NDT/SSNDT and lower-limb injuries, most often by contrasting predefined foot-type categories or applying simplified linear comparisons [17,18]. These investigations provide important evidence that altered foot mobility may be associated with injury occurrence; however, they did not model SSNDT as a continuous exposure across its full range nor examine potential non-linear risk patterns or identify specific SSNDT regions associated with minimal or elevated injury risk [18,19,20]. Both low-arched and high-arched individuals appear to be at comparable injury risk, despite systematic differences in foot mobility between these groups, further underscoring the limitations of categorical approaches [20].

From a practical standpoint, injury prevention requires more than showing that SSNDT correlates with injury occurrence. Because SSNDT may act as a bidirectional predictor, where both abnormally low and high values could be associated with elevated risk, a two-sided threshold approach anchored to a minimum-risk reference point is conceptually appropriate [14,15]. Such an approach allows SSNDT to be interpreted through progressive risk levels, analogous to clinical staging.

To date, no study has systematically examined which functional form best describes the SSNDT–injury relationship, nor derived a set of thresholds that translate the continuous SSNDT range into meaningful risk bands. Three gaps remain unresolved: (1) the optimal functional form (simple, quadratic, cubic) has not been established through formal model comparison; (2) the SSNDT value corresponding to minimum predicted risk has not been identified, limiting the ability to define a safe zone; and (3) SSNDT thresholds have not been evaluated across a dense sequence of relative-risk levels, preventing the determination of which thresholds are redundant or statistically distinct. Without such analyses, risk stratification remains arbitrary.

The novelty of this study lies in the development of a data-driven framework for characterizing a continuous SSNDT–injury risk gradient and translating it into clinically interpretable relative-risk bands along the sit-to-stand navicular drop (SSNDT) continuum. Rather than relying on traditional categorical foot-type classifications, we applied a structured modelling approach to identify a minimum-risk reference region and to define progressively higher-relative-risk bands derived from a continuous non-linear model [14].

Accordingly, the primary aim of this study was characterize the SSNDT–injury relationship as a continuous risk function and to derive clinically interpretable SSNDT-based relative-risk bands that stratify individuals from low to high injury risk. Specifically, we aimed to: (1) determine the functional form of the SSNDT–injury relationship through logistic model comparison; (2) estimate the SSNDT value associated with minimum injury risk and define a central safe zone; (3) derive model-based odds-ratio anchor levels corresponding to increasing odds-ratios and use them to construct ordered interpretive risk bands; and (4) evaluate whether the resulting band-based classification preserves the predictive structure of the underlying continuous model, rather than implying statistically discrete risk classes.

## 2. Materials and Methods

To address these aims, a stepwise analytical strategy was employed. First, we assessed whether the SSNDT–injury association was best described by a linear or non-linear logistic model. Second, the selected quadratic model was used to identify the minimum-risk region. Third, model-derived odds-ratio thresholds were used as interpretive anchors along the SSNDT continuum to derive injury risk zones, and empirical injury prevalence within these bands was quantified for descriptive and communicative purposes, rather than to imply statistically discrete risk categories.

This study focused exclusively on foot and ankle injuries in a relatively homogeneous population of physically active male university students participating recreationally in team sports. Strict inclusion and exclusion criteria were applied, as detailed below. To support stable estimation of non-linear risk patterns and model-derived thresholds, data were collected over three consecutive academic years (2023–2025), yielding three independent cohorts.

Across the three cohorts, data were collected from 59 participants in 2023, 45 participants in 2024, and 33 participants in 2025, resulting in a total sample of 137 individuals (274 feet). All assessments included anthropometric measurements, the sit-to-stand navicular drop test (SSNDT), and self-reported questionnaires assessing injury occurrence during the preceding 12 months. A subset of the data collected during the 2024 academic year has been reported previously in a separate publication addressing different research objectives [11].

To justify pooling data across cohorts, between-cohort comparability was formally evaluated. Differences in age, anthropometric characteristics, BMI, SSNDT values, and injury prevalence were examined using one-way ANOVA. In addition, Procrustes analysis was applied to assess the geometric similarity of the predicted SSNDT–injury risk curves across cohorts. Detailed statistical procedures are described in the Statistical Analysis section, with results presented in Supplementary Table S1. The findings confirmed the general equivalence of the cohorts, particularly with respect to SSNDT values and injury frequencies, supporting the decision to merge the samples into a single analytical dataset. To account for potential between-cohort variability in training exposure and sport experience, all subsequent analyses were conducted using mixed-effects logistic regression models adjusted for training load and training experience.

### 2.1. Study Design

A cross-sectional observational framework was applied. Injury occurrence was assessed using a 12-month self-reported recall questionnaire administered at the time of testing. Data collection took place in 2023, 2024, and 2025 at the Wroclaw University of Health and Sport Sciences and involved first- and second-year students enrolled in Physical Education and Sport programs.

### 2.2. Ethics

The study was approved by the Senate Research Ethics Committee of the Wroclaw University of Health and Sport Sciences (approval no. 13/2022), and all procedures adhered to the principles of the Declaration of Helsinki. Participants were informed about the purpose of the research, the assessment protocol, and data confidentiality, after which electronic informed consent was obtained prior to any data collection. Informed consent was obtained from all individual participants included in the study.

### 2.3. Sample Size

As a general planning heuristic, recommendations of 10–15 events per predictor degree of freedom (EPV) are commonly cited to ensure stable estimation in regression models including non-linear terms [21,22]. These recommendations imply a higher number of outcome events than observed in the present study [23].

The observed prevalence of foot and ankle injuries (~25%) reflects the real-world injury burden in this population and resulted in a lower absolute number of injury events. Consequently, the EPV recommendations could not be fully met and should be interpreted as approximate rather than prescriptive. To address this limitation, model stability was evaluated using mixed-effects modeling and Monte Carlo simulation-based sensitivity analyses.

For planning purposes, sample size considerations for estimating the injury proportion with a 95% confidence level and a 5 percentage-point margin of error was calculated using the standard formula [24,25]:

$$n=(\frac{1.96}{\delta }{)}^{2}\times p\left(1-p\right).$$

Assuming a conservative maximum variance (*p* = 0.50), the theoretical required sample size would be approximately 380 observations. Under a more realistic assumption of musculoskeletal injuries of ankle and foot prevalence of *p* = 0.25, the required sample size decreases to approximately 288 observations.

Because each participant contributed two feet, observations were clustered within individuals, and the effective sample size may, therefore, be lower due to intra-class correlation between left and right feet. The sample size calculations should thus be interpreted as approximate and primarily descriptive. Importantly, clustering was explicitly addressed in the primary analyses through the use of mixed-effects logistic regression models with participant-level random intercepts.

Given the deviation from conservative sample size assumptions and the clustered data structure, a Monte Carlo simulation-based sensitivity analysis was performed to evaluate the stability of model estimates and U-shaped threshold locations across three sample size scenarios (*n* = 380, *n* = 288, and *n* = 274). This analysis was intended to assess robustness of model-derived risk patterns rather than to increase statistical power. The design and results of this analysis are described in Section 2.6.2.

### 2.4. Participants

A total of 137 students were recruited across three independent data-collection periods (November–December 2023; April–May 2024; November 2025). All participants were enrolled in degree programs within the Faculty of Physical Education, Sport and Physiotherapy at the Wroclaw University of Health and Sport Sciences. Recruitment was conducted during regular classroom sessions, and participation was voluntary, with the sex distribution broadly reflecting the typical composition of these programs.

Preliminary inclusion criteria required students to be physically active and regularly attending in-person classes. To maintain homogeneity in habitual physical activity, individuals involved in university-level competitive sport or elite performance pathways were excluded. In detail, the inclusion criteria were: (1) amateur player in team game sport, (2) sport experience—at least four years of activity in team game sport, (3) regularity—at least three times a week, (4) weekly load (times per week × hours), not less than six hours/week, (5) age between 18 and 22 years. Additionally, the exclusion criteria comprised: (1) injury within the preceding three months, (2) congenital foot deformity or prior lower-limb surgery, and (3) pain that could interfere with the navicular drop assessment. Eligible participants were further required to be first- or second-year students without repeated semesters, majoring in Physical Education or Sport, engaged primarily in running or team sports, and able to provide informed consent.

Because the sit-to-stand navicular drop test (SSNDT) is a foot-specific functional measure, each foot was treated as an independent observational unit. This yielded a total of 274 analyzable feet, which formed the basis for all subsequent logistic modelling and threshold-based risk analyses.

### 2.5. Procedure

All assessments for a given participant, in each cohort, were performed during a single session. Measurements were conducted in the late morning hours (10:00 AM–12:00 PM) between academic classes. The order of measurements was identical for all individuals.

#### 2.5.1. Anthropometric Measurements

Anthropometric assessments were performed first. Body height was measured to the nearest 0.1 cm using a GPM anthropometer (GPM Instruments GmBH, Susten, Switzerland), while body mass was obtained with the InBody230 bioelectrical impedance analyzer (InBody Co., Ltd., Cerritos, CA, USA). Body mass index (BMI) was calculated using the following formula:

$$BMI\;[kg/{cm}^{2}]=bodyweight\;[kg]/body{height}^{2}\;[m^{2}].$$

#### 2.5.2. Recording Injuries—Injury History Questionnaire (IHQ)

Musculoskeletal injuries were recorded to calculate injury frequency, with a focus on foot and knee injuries. The Injury History Questionnaire (IHQ) was utilized, and a detailed description of this tool has been presented elsewhere [26]. The authors assessed the IHQ as a reliable and valuable instrument, which was confirmed by a Cronbach’s alpha coefficient of 0.836.

#### 2.5.3. Sit-To-Stand Navicular Drop Test

In this study, a modified protocol—the sit-to-stand navicular drop test (SSNDT)—was employed [14]. It is modification of the test originally described by Brody [15]. The SSNDT quantifies the displacement of the navicular bone by measuring the change in navicular height (NH) between a subtalar neutral position in sitting and a weight-bearing position in standing. Based on previously established cut-off values, foot posture is typically classified as supinated (<5 mm), neutral (5–9 mm), or pronated (10–15 mm) [14].

Measurement reliability of the SSNDT has been evaluated previously by the present author in an independent sample, with the results reported elsewhere [7]. Within-day test–retest reliability was assessed using a two-way mixed-effects intraclass correlation coefficient model (ICC(3,1)), demonstrating good reproducibility (ICC(3,1) = 0.82, 95% CI: 0.55–0.919, F = 9.18, *p* < 0.0001).

During data collection, participants were assessed barefoot in a seated position. The navicular tuberosity of each foot was identified by palpation and marked using a hypoallergenic marker. Subtalar neutral alignment was established manually. A rigid paper card was placed against the medial aspect of the foot, and the vertical distance from the floor to the marked navicular tuberosity was measured with a ruler. Participants then assumed a standing position, with one foot placed on a calibrated medical scale and the contralateral foot supported on a platform of equal height. They were instructed to transfer approximately 80% of their body weight onto the tested foot. The navicular height in standing was marked on the card, and the SSNDT value was calculated as the difference between seated and standing measurements.

Previous investigations have demonstrated that the SSNDT exhibits superior measurement reliability (ICC(3,1) range: 0.73–0.96) compared with the traditional NDT procedure (ICC(3,1) range: 0.37–0.68) [14]. In this study, foot posture classification followed Brody’s original criteria [15]. Based on bilateral SSNDT values, participants were categorized into three groups: neutral alignment in both feet, mixed alignment (one neutral and one non-neutral foot), or non-neutral alignment in both feet (supinated or pronated). This categorical variable was subsequently incorporated as a predictor in multiple logistic regression analyses.

### 2.6. Statistical Analysis

#### 2.6.1. Validation the Datasets Consistency

Between-dataset differences in age, somatic characteristics, and SSNDT were assessed using one-way ANOVA. No significant differences were observed for age, anthropometrical measurements nor SSNDT (*p* > 0.05) (Supplementary Table S1). However, there were statistically significant differences in sport activity experience and weekly load (ANOVA: F = 26.57, *p* < 0.001; F = 8.16, *p* < 0.001, respectively). Post hoc tests with Bonferroni correction revealed significantly longer experience and weekly load in the cohort studied in 2023 than older cohorts (difference in experience was about 0.3 year, while in load about 1 h/week). Importantly, injury prevalence did not differ significantly between cohorts (χ^2^ = 1.16, *p* = 0.559), suggesting comparable outcome distributions despite differences in training exposure.

To further evaluate whether cohort membership modified the SSNDT–injury association, training experience and weekly training load were subsequently included as covariates in all mixed-effects logistic regression models. This adjustment allowed the SSNDT–injury relationship to be examined independently of cohort-related differences in training exposure. Descriptive statistics and between-dataset comparisons, including ANOVA and post-hoc tests, are provided in Supplementary Table S1.

#### 2.6.2. Monte Carlo Synthetic Data Simulation

To evaluate the robustness of the estimated U-shaped SSNDT–injury relationship and associated model-derived thresholds, a Monte Carlo simulation-based sensitivity analysis was conducted. Synthetic datasets were generated to reflect the observed distribution of SSNDT values and the expected non-linear relationship between SSNDT and ankle and foot injury risk, with minimal risk occurring within the literature-supported normative range and progressively increasing risk toward both lower and higher extremes.

Simulation scenarios were designed to reflect realistic injury prevalences and SSNDT distributions observed in the empirical dataset, rather than to estimate statistical power. For each scenario, a large number of synthetic datasets were generated, and identical quadratic logistic regression models were fitted to each replicate. Model-derived thresholds, predicted risk curves, and selected performance metrics were extracted and summarized across simulations.

Across simulation scenarios, the shape of the risk curve and the relative ordering of threshold estimates were stable and closely aligned with those observed in the empirical data, supporting the robustness of the modeled SSNDT–injury relationship.

#### 2.6.3. Model Comparison and Functional-Form Evaluation

To identify a single, well-defined minimum-risk region and derive two interpretable risk thresholds on either side of this minimum, the functional form of the SSNDT–injury association was formally evaluated. Nested mixed-effects logistic regression models with increasing functional complexity were fitted, including linear, quadratic, and cubic specifications of SSNDT. All models included a random intercept for participant ID to account for within-subject dependence between bilateral feet and were adjusted for training load and training experience. Model fit and parsimony were compared using the Akaike Information Criterion (AIC), Bayesian Information Criterion (BIC), Nagelkerke’s pseudo-R^2^, and likelihood-ratio tests for nested models.

Although the cubic specification was formally evaluated and showed improved global fit indices, it introduced multiple inflection points and did not yield a single, stable minimum-risk region. Given the study objective of deriving interpretable two-sided thresholds, the cubic model was not retained. The quadratic functional form was, therefore, selected as the most parsimonious and clinically interpretable representation of the SSNDT–injury relationship and served as the basis for all subsequent threshold and risk-zone analyses.

#### 2.6.4. Identification of the Minimum-Risk Point

For the quadratic model, the SSNDT value corresponding to the minimum predicted injury probability was obtained analytically from the fixed-effect estimates of the mixed-effects logistic regression model. This value served as the reference point (OR = 1.0) for all subsequent threshold computations and defined the center of the “safe zone”.

Given a quadratic functional form with a positive second-order coefficient, the location of the minimum-risk point was derived analytically as:

$${SSNDT}_{min}=-\frac{\beta_{1}}{2\beta_{2}}$$

where *β*_1_ and *β*_2_ denote the fixed-effect coefficients for SSNDT and SSNDT^2^, respectively.

Based on the fixed-effect estimates, the minimum-risk SSNDT value was 5.5 mm. This indicates that foot mobility close to this level was associated with the lowest predicted probability of injury. SSNDT values both below and above this central region corresponded to progressively increasing injury risk, forming the basis for subsequent odds-ratio-based threshold estimation.

#### 2.6.5. Threshold Estimation Across a Dense OR Grid

To map SSNDT values onto progressive levels of relative risk, thresholds were calculated for odds-ratio (OR) levels ranging from 1.1 to 2.0 in 0.1 increments based on predictions from the adjusted mixed-effects quadratic logistic regression model. For each OR target, two SSNDT values (lower and upper) satisfying:

$$OR\left(SSNDT\right)={OR}_{target}$$

were obtained using numerical root-finding while holding all covariates at their mean values. This procedure yielded a sequence of potential two-sided thresholds representing increasingly elevated injury risk.

#### 2.6.6. Bootstrap Evaluation of Threshold Distinguishability

To determine which thresholds represented statistically distinct changes in SSNDT-based risk, nonparametric bootstrap resampling (1000 iterations) was applied at the participant level. In each bootstrap iteration, the quadratic mixed-effects logistic regression model was refitted, the minimum-risk point was recalculated, and the pair of lower and upper thresholds were recomputed for each OR level (1.1–2.0). Adjacent OR-based thresholds were classified as: (1) non-redundant (distinct, non-overlapping confidence intervals for both lower and upper thresholds), or (2) redundant (overlapping confidence intervals and similar SSNDT ranges), indicating statistically indistinguishable threshold locations.

Because substantial overlap of confidence intervals was observed across adjacent OR levels, thresholds were not interpreted as discrete statistical breakpoints. Instead, selected OR levels were retained as clinically interpretable anchor points along a continuous risk gradient and were subsequently used to construct SSNDT-based risk zones.

#### 2.6.7. Segmented Regression and Local Risk Gradients Estimation

To investigate whether the SSNDT–injury association contained any discrete change-points beyond the minimum-risk region, segmented logistic regression models with up to two breakpoints were fitted using the SSNDT predictor as a continuous variable. All segmented models were fitted within the same adjusted mixed-effects framework, including a random intercept for participant ID and adjustment for training load and training experience. Breakpoint estimates, their standard errors, and changes in model deviance and information criteria were used to evaluate the presence and stability of potential additional thresholds along the SSNDT continuum. In parallel, the local risk gradient was characterized by deriving the first derivative of the quadratic logit function, β′(x), based on the fixed-effect estimates, and converting it to a local odds-ratio associated with a 1 mm increase in SSNDT at selected values across the observed range. This analysis provided a pointwise description of how rapidly injury risk increased as SSNDT deviated from its minimum-risk value and allowed direct comparison with the absence of discrete breakpoints identified by segmented regression.

#### 2.6.8. Derivation and Evaluation of SSNDT-Based Risk Zones

SSNDT values were categorized into ordered risk zones (Safe, Mild, Moderate, High, and Extreme) using selected anchor odds-ratio (OR) levels (1.2, 1.5, 1.8, and 2.0) derived from the adjusted quadratic mixed-effects model. These zones were defined as interpretive segments along a continuous risk gradient rather than as statistically discrete risk classes.

Their predictive performance was assessed using sensitivity, specificity, positive and negative predictive values, and overall classification accuracy, derived from a confusion matrix contrasting the combined high/extreme zones against all lower-risk categories. This dichotomization was chosen to reflect a pragmatic screening scenario focused on identifying individuals at clearly elevated risk.

Performance metrics derived from the zone-based classification were interpreted descriptively and contrasted with predictions from the continuous quadratic model to illustrate how discretization of a continuous risk function affects classification characteristics, rather than to establish superiority over the continuous model.

#### 2.6.9. SSNDT Rater Reliability Validation—Intraclass Correlation Coefficient (ICC)

Intra-rater within-day test-retest reliability of SSNDT measurements were evaluated using the intraclass correlation coefficient model ICC(3,1), according to the Shrout and Fleiss convention [27]. This specification corresponds to a two-way mixed-effects model with absolute agreement and single-measure reliability [28]. Reliability was calculated based on two successive SSNDT measurements obtained from nine participants (18 feet). ICC values were interpreted according to Koo and Li [28] as follows: <0.50 poor, 0.50–0.75 moderate, 0.75–0.90 good, and >0.90 excellent reliability.

All analyses were conducted in Statistica 14.0 (TIBCO Software Inc., Palo Alto, CA, USA) and RStudio (2025.09). Continuous variables were inspected for normality (Shapiro–Wilk test) and homogeneity of variance (Levene’s test). Descriptive statistics are reported as means ± SD with 95% confidence intervals or as frequencies (%). Between-group comparisons used independent *t*-tests (continuous variables) or chi-square tests (categorical variables). Statistical significance was set at *p* < 0.05. Graphical visualizations of the SSNDT–injury risk curve, local risk gradients, and OR-based thresholds were created using ggplot2 in R.

#### 2.6.10. AI Transparency Statement

Generative AI tools were used in accordance with COPE and MDPI transparency principles and were limited to preparatory, editorial, and technical support. Chat Academia (v1.0, 2025) assisted in refining research questions and identifying potential gaps, while Scholarcy (v4.0, 2025) and NotebookLM (v1.3, 2025) were used for preliminary literature summaries and note-taking. Elicit (v2.0, 2025) supported semantic literature searches only. The statistical analysis framework was developed independently by the authors; however, Julius.ai platform (2025) and ChatGPT (OpenAI, GPT-4.1, 2025) were used to support the statistical workflow by facilitating access to documentation for R (v. 4.5.2) packages (e.g., CRAN Task Views (v. 0.9-7)) and methodological references. Minor R coding issues were resolved using AI-assisted diagnostic tools within RStudio, with full manual verification. During manuscript preparation, ChatGPT was used for language editing. All AI-assisted content was reviewed and approved by the authors, who take full responsibility for the final manuscript.

## 3. Results

The Results section is structured to reflect the sequential analytical workflow. We first present model selection and shape assessment of the SSNDT–injury association, followed by estimation of the minimum-risk point and derivation of odds-ratio-based thresholds. Subsequently, injury prevalence across the resulting SSNDT zones is reported, and the robustness of these findings is evaluated using Monte Carlo simulations.

### 3.1. Intra-Rater Reliability—ICC Analysis

Intra-rater within-day reliability of SSNDT measurements was assessed using an ICC(3,1) model. Reliability was evaluated based on two successive measurements obtained from nine participants (18 feet). The resulting intra-rater reliability was classified as good (ICC(3,1) = 0.82, 95% CI: 0.55–0.92, F = 9.18, *p* < 0.001).

### 3.2. Participant Characteristics

Baseline characteristics are presented in Table 1. Descriptive data are shown as means ± standard deviations with 95% confidence intervals for the means. Anthropometric characteristics did not differ significantly between not-injured and injured males (all *p* > 0.05), whereas a significant difference was observed for SSNDT (*p* < 0.001). Out of the 274 assessed feet, 58 (21.2%) suffered at least one injury of ankle or foot during the year preceding the study, while 216 (78.8%) did not.

### 3.3. Comparison of Logistic Model Functional Forms

Three nested mixed-effects logistic regression models with increasing functional complexity were fitted to characterize the association between SSNDT and injury occurrence: a linear model, a quadratic model including SSNDT and SSNDT^2^, and a cubic model additionally including SSNDT^3^. All models incorporated a random intercept for participant ID and were adjusted for training load and training experience.

As shown in Table 2, model fit indices improved with increasing functional complexity. Both AIC and BIC decreased from the linear to the quadratic specification, with a further decrease observed for the cubic model (AIC: 247.59, 239.80, and 214.46; BIC: 265.65, 261.48, and 239.75 for the linear, quadratic, and cubic models, respectively). Similarly, Nagelkerke’s pseudo-R^2^ increased across models (0.227, 0.274, and 0.396).

Despite the improved goodness-of-fit metrics for the cubic model, visual inspection and analytical evaluation indicated that it introduced multiple local extrema and did not yield a single, well-defined minimum-risk region. In contrast, the quadratic specification provided a clear and interpretable U-shaped relationship with a single minimum-risk point. Therefore, the quadratic model was selected as the most parsimonious and conceptually appropriate representation of the SSNDT–injury association and served as the basis for all subsequent threshold and risk-zone analyses.

### 3.4. Identification of the Minimum-Risk SSNDT Value: Quadratic Model Fit and U-Shaped Risk Structure

Table 3 presents the fixed-effect parameter estimates from the adjusted mixed-effects quadratic logistic regression model. The linear SSNDT term was positive (for mean-centered SSNDT), whereas the quadratic term was also positive, indicating a U-shaped relationship between SSNDT and injury probability with a single minimum-risk region.

Based on the fixed-effect estimates, the minimum-risk SSNDT value was 5.5 mm. This indicates that foot mobility close to this level was associated with the lowest predicted probability of injury. SSNDT values both below and above this central region corresponded to progressively increasing injury risk, forming the basis for subsequent odds-ratio-based threshold estimation. The minimum-risk point (OR = 1.0) served as the reference for all two-sided SSNDT thresholds in the following analyses.

### 3.5. OR-Based Threshold Estimation Across a Dense Risk Grid

Using the quadratic mixed-effect logistic model and the minimum-risk value identified in Section 3.4 (SSNDT_min_ = 5.5 mm), two-sided thresholds were calculated for a series of increasing odds-ratio (OR) levels. For each target OR between 1.1 and 2.0 (in 0.1 increments), we numerically identified the SSNDT values at which the predicted odds of injury reached the specified relative-risk level compared with the minimum-risk point (OR = 1.0).

Each OR level yielded two thresholds: a lower value on the left arm of the U-shaped curve (reduced foot mobility) and an upper value on the right arm (excessive mobility). As shown in Table 4, thresholds moved progressively away from the minimum-risk point as the OR increased. The lower thresholds shifted from 4.12 mm at OR = 1.1 to 1.80 mm at OR = 2.0, whereas the upper thresholds increased from 6.85 mm to 9.17 mm across the same OR range.

This pattern reflects an expanding region of elevated injury risk at both extremes of the SSNDT continuum, with asymmetric widening consistent with the curvature of the quadratic model. These model-derived thresholds provide the basis for subsequent analyses examining the statistical distinguishability and practical interpretation of increasing SSNDT-related injury risk.

### 3.6. Bootstrap Distinguishability of Adjacent OR Thresholds

While Section 3.5 provided the model-derived SSNDT thresholds for a sequence of OR values, Section 3.6 evaluates whether these thresholds represent statistically distinct risk levels or simply reflect smooth, continuous changes along the underlying curve.

To assess whether successive odds-ratio (OR) levels produced distinguishable SSNDT thresholds, a nonparametric bootstrap procedure (1000 resamples) was applied. In each bootstrap iteration, the quadratic mixed-effects model was refitted, the minimum-risk point was recalculated, and SSNDT thresholds corresponding to OR = 1.1–2.0 were re-estimated. This procedure yielded bootstrap-based 95% confidence intervals for both the lower and upper thresholds at each OR level.

Adjacent OR levels were classified as distinct when their 95% confidence intervals for both thresholds did not overlap, and as redundant when intervals overlapped, indicating that the corresponding SSNDT values were statistically indistinguishable. The complete bootstrap results are shown in Table 5.

Overlap of bootstrap confidence intervals across adjacent OR levels indicated the absence of discrete breakpoints, consistent with a continuous risk gradient. Across the entire OR range (1.1–2.0), all adjacent OR levels exhibited substantial overlap in their bootstrap confidence intervals for both the lower and upper thresholds. This pattern demonstrates that the SSNDT–injury relationship evolves in a smooth, continuous manner, without discrete or abrupt changes in the threshold structure between closely spaced OR levels. As a result, none of the intermediate OR increments (e.g., 1.2 → 1.3, 1.4 → 1.5) produced statistically distinct thresholds.

Consequently, the subsequent formation of practical SSNDT risk zones was based not on statistical separation between adjacent OR levels, but on clinically interpretable anchor points representing meaningful relative-risk increases (e.g., OR ≈ 1.2, 1.5, 2.0). These OR-based thresholds should, therefore, be interpreted as representative markers along a continuous risk gradient rather than as strict statistical cut-points.

### 3.7. Segmented Regression and Local Risk Gradients

Segmented regression analysis was used to examine whether the SSNDT–injury association exhibits discrete changes in slope beyond the minimum-risk region identified by the quadratic model. A one-breakpoint segmented model identified a single statistically significant change in slope at ψ ≈ 5.1 mm (Davies’ test *p* < 0.001), located within the vicinity of the minimum-risk region estimated from the quadratic model (Supplementary Table S2). This breakpoint does not represent an additional risk transition but reflects the turning point of the U-shaped relationship. The one-breakpoint model improved fit compared with a simple linear specification, whereas a two-breakpoint model showed clear signs of numerical instability and overfitting and did not yield interpretable or reproducible breakpoint estimates.

**Figure 1:**
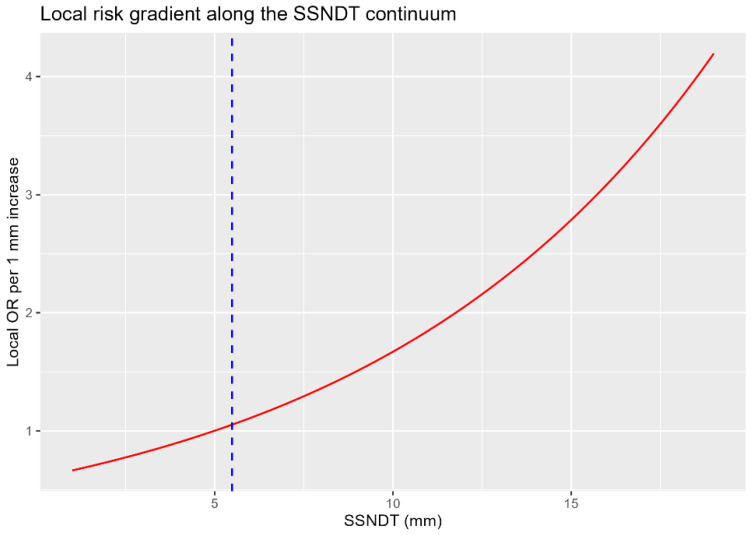
Continuous local risk gradient along the SSNDT continuum. The local odds-ratio associated with a 1 mm increase in SSNDT was derived from the quadratic logistic regression model (fixed-effects component). The dashed vertical line marks the minimum-risk point (SSNDT_min_ ≈ 5.5 mm). The smooth, monotonic rise in the local OR with increasing deviation from SSNDT_min_ demonstrates the absence of additional breakpoints and supports continuous—rather than stepwise—risk progression.

Analysis of the local risk gradient derived from the quadratic model demonstrated a smooth and progressive increase in injury risk as SSNDT deviated from the minimum-risk point, with no evidence of additional discrete transitions (Figure 1). Together, these findings support interpretation of the SSNDT–injury relationship as a continuous risk gradient rather than a stepwise process, with the segmented breakpoint effectively capturing the same minimum-risk location identified analytically by the quadratic model.

Taken together, the segmented and gradient analyses indicate that the only detectable change in slope corresponds to the minimum-risk region itself, with no evidence of additional secondary breakpoints along the SSNDT continuum. Consequently, practical risk stratification must rely on model-derived relative-risk increments applied to a continuous risk function rather than empirically detected step transitions. This provides a methodological rationale for the subsequent derivation of OR-based SSNDT thresholds and the construction of clinically interpretable risk zones.

### 3.8. Construction of SSNDT Risk Zones

Based on the selected odds-ratio (OR) anchor levels identified through the bootstrap uncertainty analysis (OR = 1.2, 1.5, 1.8, and 2.0), four SSNDT-based risk zones were constructed. These zones (Table 6) define progressively wider regions of elevated injury risk as SSNDT deviates from the minimum-risk point (≈5.5 mm).

The mild-risk zone (OR = 1.2) spans approximately 3.6–7.4 mm, representing relatively small deviations from the central minimum-risk region. The moderate-risk zone (OR = 1.5) extends further (2.7–8.3 mm), while the elevated-risk (OR = 1.8; 2.1–8.9 mm) and high-risk (OR = 2.0; 1.8–9.2 mm) zones correspond to increasingly distant SSNDT values on both sides of the U-shaped risk curve.

Together, these model-derived zones provide an interpretable mapping of the continuous SSNDT–injury risk function into clinically communicable ranges for screening and individual risk profiling. Importantly, the zones are intended as descriptive anchors along a continuous risk gradient rather than as statistically discrete cut-points. In the next section, we evaluate their classification performance and the degree of information retained when moving from a continuous predictor to a zone-based representation.

### 3.9. Translation of Continuous SSNDT Risk into OR-Based Bands and Interpretive Risk Zones

**Figure 2:**
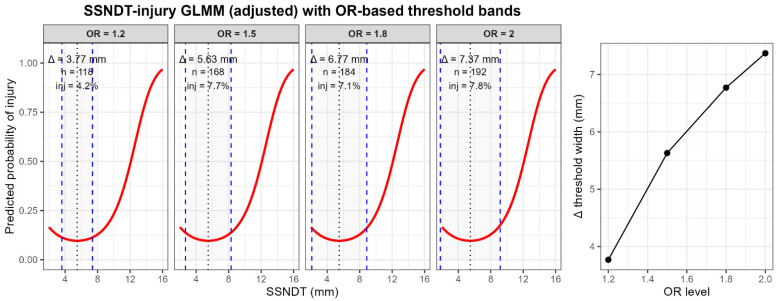
Quadratic SSNDT–injury relationship with OR-based threshold bands (OR = 1.2, 1.5, 1.8, 2.0). Numbers displayed between thresholds indicate the total number of observations and empirical injury prevalence within each SSNDT band. Because OR-based bands widen symmetrically away from the minimum-risk point, sample sizes increase across higher OR levels, while the underlying model-based risk increases monotonically. Dotted vertical lines indicate the SSNDT value associated with the minimum predicted injury risk derived from the fitted GLMM model. Dashed vertical lines represent the lower and upper SSNDT thresholds corresponding to the specified odds ratio (OR) levels, defining the width of each OR-based threshold band (Δ).

Based on the continuous quadratic mixed-effects model, injury risk increased monotonically as SSNDT deviated from the minimum-risk point. To translate this continuous risk function into applied decision support, OR-based threshold bands (OR = 1.2, 1.5, 1.8, and 2.0) were defined and subsequently labelled as interpretive SSNDT risk zones. The resulting model-based risk structure is illustrated in Figure 2.

When empirical injury counts were aggregated within these discretized SSNDT zones, some local non-monotonic variation in injury prevalence was observed. In particular, the Moderate zone showed no injuries, while the Mild zone demonstrated a higher prevalence than the Safe zone. Importantly, this pattern reflects descriptive aggregation within discretized intervals rather than properties of the underlying risk function. These local irregularities likely reflect small sample sizes in intermediate categories and the proximity of these zones to the minimum-risk region of the continuous U-shaped SSNDT–injury relationship (Figure 2), rather than true departures from a graded risk structure.

To assess practical screening performance, the High and Extreme zones were combined as a positive classification for elevated injury risk. This approach yielded good sensitivity (0.78) and specificity (0.79), with a high negative predictive value (0.93), indicating effective identification of individuals at clearly elevated risk while reliably excluding those at low risk. Thus, while empirical injury proportions within individual zones are not expected to follow strict monotonic ordering, the zone-based classification preserves the monotonic structure of the underlying continuous risk model and provides a clinically interpretable summary for applied risk stratification (Table 7).

## 4. Discussion

In this study, we evaluated the sit-to-stand navicular drop test (SSNDT) as a continuous predictor of foot and ankle injury risk and demonstrated that the SSNDT–injury association follows a non-linear, U-shaped pattern with a clearly identifiable minimum-risk region. Sensitivity analyses based on Monte Carlo simulations showed that, under realistic injury prevalence (~20%), the empirical data closely aligned with simulated scenarios, supporting the adequacy of the achieved sample size for non-linear modelling.

These findings provide a data-driven foundation for characterizing a continuous, model-based relationship between foot mobility and injury risk, which can be translated into clinically interpretable risk bands for applied use. Across the predefined aims, the results consistently supported the proposed framework. The quadratic model provided a more appropriate representation than linear alternatives; the minimum-risk SSNDT value was estimated at approximately 5.5 mm; and injury risk increased smoothly across the SSNDT continuum without evidence of discrete secondary breakpoints. A limited set of representative odds-ratio anchor levels (OR = 1.2, 1.5, 1.8, and 2.0) was used to derive interpretative risk bands, which preserved the main predictive structure of the continuous model and showed high specificity for identifying low-risk individuals.

Foot mobility has long been recognized as a potential intrinsic risk factor for lower-limb injuries [29,30,31,32], yet commonly used classifications of pronated, neutral, and supinated foot types still rely on arbitrary cut-points rather than empirical risk patterns [33,34]. In this study, we evaluated the sit-to-stand navicular drop test (SSNDT) as a continuous predictor of injury risk and demonstrated that the SSNDT–injury relationship follows a non-linear, U-shaped pattern with a clearly identifiable minimum-risk region. This provides a data-driven foundation for modeling the continuous relationship between foot mobility and injury risk, from which model-based relative-risk thresholds can be derived to facilitate clinical interpretation compared with traditional foot-type assessments [35,36].

Consistent with our first aim, the quadratic logistic model outperformed both linear and cubic models, confirming earlier work suggesting that foot mobility exhibits a curvilinear association with injury susceptibility [37,38]. The estimated minimum-risk value (~5.5 mm) aligns with previous research indicating that both excessive pronation and excessive supination may elevate injury risk due to altered load distribution across the medial longitudinal arch and subtalar joint [20,39,40]. These observations are also in line with evidence showing that broader biomechanical characteristics—including postural alignment and structural factors—modulate injury susceptibility [41,42]. Runners with mobile arches exhibit different lower extremity movement patterns and forces compared to those with rigid arches [43].

The dense grid of relative-risk estimates (OR 1.1–2.0) revealed that risk increases smoothly and continuously as SSNDT departs from its optimal value, with no discrete empirical breakpoints, a finding supported by both bootstrap uncertainty analysis and segmented regression. This is consistent with contemporary biomechanical models positing that foot mobility influences tissue loading gradients rather than acting as a categorical risk determinant [44,45]. Similar conclusions were drawn in our previous work, where continuous predictors such as body composition indices outperformed categorical classifications in identifying musculoskeletal injury risk [46].

By selecting a limited set of clinically interpretable OR anchor levels (OR = 1.2, 1.5, 1.8, 2.0), we translated the continuous SSNDT–injury risk function into interpretive risk bands spanning increasing deviations from the minimum-risk region. This offers a more nuanced risk-stratification framework compared with traditional classifications, which typically rely on single cut-offs (e.g., NDT > 10 mm) that may oversimplify the variability in foot mechanics [4,47]. Although sensitivity of the zone-based classification was modest, specificity was high, suggesting strong utility for ruling out low-risk individuals. Importantly, these bands were introduced as communication and screening aids rather than statistically separable risk classes. This pattern mirrors findings from other screening tools in sports medicine, where specificity often exceeds sensitivity in identifying intrinsic risk profiles [20,48,49]. From an applied perspective, these results complement previous findings linking movement-quality characteristics with performance and injury-related biomechanical deficits [42].

It should be noted that the Mild-Moderate zone exhibited no observed injuries; however, this finding was based on a relatively small number of observations (*n* = 16). As such, the absence of injuries in this zone should not be interpreted as evidence of zero risk, but rather as a lack of observed events within a limited sample. Small cell sizes reduce the precision of zone-specific injury prevalence estimates and increase uncertainty around empirical risk classification. Importantly, the designation of this zone as “safe” is primarily based on the model-estimated minimum-risk region of the continuous SSNDT–injury curve rather than on raw injury counts alone. Therefore, the “safe” label should be interpreted as reflecting a relative minimum of modeled risk rather than an absolute injury-free category.

This study has several notable strengths. First, it introduces a data-driven framework for interpreting the SSNDT by modeling a continuous injury risk gradient, moving beyond traditional categorical foot-type assessments based on arbitrary cut-points. Second, the modelling strategy was comprehensive, combining model comparison, quadratic risk estimation, dense OR-based threshold mapping, segmented regression, and bootstrap uncertainty quantification. This multilayered approach allowed precise identification of the minimum-risk region and robust evaluation of whether empirical breakpoints exist along the SSNDT continuum. Third, translating continuous model predictions into clinically interpretable risk bands provides practitioners with actionable screening tools that retain most of the predictive information of the underlying continuous model. Finally, the sample size of 274 feet provides sufficient statistical power to characterize the SSNDT–injury relationship and derive stable, reproducible thresholds.

The OR-based SSNDT bands offer a practical framework for interpreting foot mobility as a continuous injury-risk indicator rather than a binary classification. By referencing a model-derived minimum-risk region, both low and high SSNDT values can be interpreted within a graded risk continuum. The framework is particularly useful for screening purposes, as the High and Extreme bands identify individuals at clearly elevated risk while maintaining high specificity for ruling out low-risk cases. Importantly, intermediate bands should be viewed as descriptive risk markers reflecting gradual changes in predicted risk rather than discrete diagnostic thresholds.

Several limitations should be considered. First, although the study was conducted within a prospectively approved observational research framework, injury occurrence was assessed using a 12-month self-reported recall questionnaire administered at a single time point. This limits causal inference and may introduce recall bias. Such bias may affect the absolute number of reported injuries, particularly minor events; however, because it is unlikely to be systematically related to SSNDT values, its primary effect would be to attenuate associations rather than distort the overall non-linear structure of the SSNDT–injury relationship or the location of the minimum-risk (“safe”) zone. Second, although the sample included a relatively large number of feet, the study recruited only young adult males, restricting the generalizability of the thresholds to females, adolescents, or older populations. Third, the quadratic model provides an excellent statistical fit, but more complex non-linear structures—such as splines or generalized additive models—were not explored and may capture subtler deviations in populations with different foot characteristics. Fourth, although the classification performance of the risk zones was acceptable, sensitivity remained modest, indicating that zone-based categorization does not capture all high-risk individuals. Fifth, although the observed number of injury events was lower than commonly recommended EPV heuristics, this reflects the underlying injury prevalence in the studied population rather than selective under-sampling. The limited number of events may increase uncertainty in parameter estimates; however, the robustness of the quadratic functional form and derived risk thresholds was supported by mixed-effects modeling and simulation-based sensitivity analyses. Lastly, injury type, severity, and training exposure were not stratified, and these factors may influence the relationship between SSNDT and injury risk.

## 5. Conclusions

This study demonstrates that the SSNDT–injury relationship is best represented by a non-linear, U-shaped model with a clearly defined minimum-risk value. The absence of additional empirical breakpoints along the SSNDT continuum supports interpretation of injury risk as a continuous gradient, from which a limited set of clinically interpretable odds-ratio-based anchor thresholds were derived. These thresholds define practical SSNDT risk zones that exhibit an overall increasing trend in injury prevalence and retain high specificity for identifying low-risk individuals. Overall, SSNDT provides a robust quantitative tool for assessing foot mobility and its association with injury risk, offering an evidence-based alternative to traditional categorical foot-type classifications. Future studies should validate these thresholds in broader populations and examine their predictive utility in prospective injury-surveillance designs.

## Figures and Tables

**Table 1:** Descriptive statistics of anthropometric measurements in the whole group and in the not injured and injured subgroups.

Measurement	Overall (*n* = 274)				Not Injured (*n* = 216)				Injured (*n* = 58)				
	Mean	95%CI		SD	Mean	95%CI		SD	Mean	95%CI		SD	*p*
Age [y]	20.5	20.4	20.6	1.0	20.4	20.3	20.6	1.0	20.4	20.7	20.4	20.9	0.121
Body height [cm]	185.9	185.1	186.7	6.9	186.0	185.1	187.0	7.1	186.0	185.6	184.0	187.2	0.705
Body weight [kg]	80.6	79.7	81.6	8.0	80.6	79.6	81.7	7.8	80.6	80.6	78.4	82.9	0.988
BMI [kg/m^2^]	23.3	23.1	23.5	1.5	23.3	23.1	23.5	1.4	23.3	23.4	22.9	23.8	0.666
SSNDT [mm]	7.8	7.4	8.2	3.0	7.2	6.9	7.5	2.5	10.1	9.2	11.1	11.1	**<0.001**
Load [h/week]	7.7	7.4	8.1	3.1	7.7	7.3	8.1	3.1	7.7	7.8	7.0	8.6	0.899
Experience [years]	4.3	4.2	4.4	0.6	4.3	4.2	4.3	0.6	4.3	4.4	4.2	4.6	0.155

Footnote: BMI—body mass index, SSNDT—sit-to-stand navicular drop test, CI—confidence interval, SD—standard deviation; significant difference was marked with bold font.

**Table 2:** Comparison of logistic regression models with alternative functional forms of the SSNDT–injury relationship (*n* = 274 feet).

Model	AIC	BIC	Nagelkerke R^2^
Linear	247.59	265.65	0.227
Quadratic	239.80	261.48	0.274
Cubic	214.46	239.75	0.396

**Table 3:** Fixed-effect parameter estimates from the adjusted mixed-effects quadratic logistic regression model predicting injury occurrence from SSNDT (*n* = 274 feet, 137 participants).

Parameter	Estimate	Std. Error	z-Value	*p*-Value
Intercept	−1.960	0.229	−8.57	<0.001
SSNDT (β_1_)	0.238	0.059	4.04	<0.001
SSNDT^2^ (β_2_)	0.051	0.017	3.06	0.002
Training load	0.005	0.055	0.10	0.921
Training experience	0.341	0.240	1.42	0.156
Model fit indices: Residual deviance = 228.54; AIC = 240.54; BIC = 262.22 Nagelkerke R^2^ = 0.279				

Note: SSNDT—sit-to-navicular drop test; SSNDT, training load, and training experience were mean-centered prior to analysis. Fixed-effect estimates are reported.

**Table 4:** Model-derived SSNDT thresholds corresponding to increasing odds-ratio (OR) levels relative to the minimum-risk point.

OR Target	SSNDT_lower_ (mm)	SSNDT_upper_ (mm)
1.1	4.12	6.85
1.2	3.60	7.37
1.3	3.22	7.75
1.4	2.92	8.05
1.5	2.67	8.30
1.6	2.45	8.52
1.7	2.26	8.71
1.8	2.10	8.87
1.9	1.94	9.03
2	1.80	9.17

**Table 5:** Bootstrap-based SSNDT thresholds and 95% confidence intervals (CI) for increasing odds-ratio levels (OR 1.1–2.0) relative to the minimum-risk point (SSNDT_min_ = 5.5 mm). The last column indicates whether thresholds are statistically redundant compared with the previous OR level (overlapping 95% CIs for both lower and upper thresholds).

OR Target	Lower	Lower 95% CI (mm)	Upper	Upper 95% CI (mm)	Redundant vs. Previous OR
1.1	4.12	1.49–5.78	6.85	3.66–8.06	-
1.2	3.60	1.37–5.36	7.37	4.43–8.53	Yes
1.3	3.22	1.30–5.06	7.75	5.02–8.87	Yes
1.4	2.92	1.21–4.84	8.05	5.48–9.13	Yes
1.5	2.67	1.18–4.65	8.30	5.85–9.36	Yes
1.6	2.45	1.12–4.48	8.52	6.17–9.59	Yes
1.7	2.26	1.14–4.34	8.71	6.42–9.76	Yes
1.8	2.10	1.12–4.20	8.87	6.66–9.93	Yes
1.9	1.94	1.11–4.08	9.03	6.89–10.08	Yes
2	1.80	1.09–3.98	9.17	7.06–10.21	Yes

**Table 6:** Model-derived SSNDT threshold bands based on selected odds-ratio levels relative to the minimum-risk point.

OR Level	OR	Lower Threshold (mm)	Upper Threshold (mm)	95%CI Lower	95%CI Upper
OR = 1.2 band	1.2	3.60	7.37	1.39–5.34	4.97–8.53
OR = 1.5 band	1.5	2.67	8.30	1.18–4.60	6.23–9.40
OR = 1.8 band	1.8	2.10	8.87	1.19–4.17	6.95–9.96
OR = 2.0 band	2	1.80	9.17	1.12–3.94	7.32–10.24

**Table 7:** Distribution of SSNDT risk zones and injury prevalence.

Risk Zone	*n*	Injured (*n*)	Injured (%)
Safe	118	5	4.2%
Mild	50	8	16.0%
Moderate	16	0	0.0%
High	8	2	25.0%
Extreme	82	43	52.4%

Note: Percentages represent empirical injury prevalence within discretized SSNDT intervals. Risk zones are interpretive labels applied to OR-based threshold bands and are not expected to strictly show monotonic injury prevalence.

## Data Availability

The data presented in this study are available upon request from the author.

## Supplementary Materials

The following supporting information can be downloaded at: https://www.mdpi.com/article/10.3390/jcm15031027/s1, Table S1: Descriptive statistics of the age, anthropometric measurements, BMI, SSNDT, experience and weekly load of training in three independent cohorts of the students. One-way ANOVA test results; Table S2: Continuous SSNDT risk gradient and structural diagnostics from segmented regression.
