# Supplementary material for: Sit-to-Stand Navicular Drop Test-Based Injury Risk Zones Derived from a U-Shaped Relationship in Male University Athletes
Source: J Clin Med. 2026 Jan 27;15(3):1027. doi: 10.3390/jcm15031027 (PMC12898812; doi:10.3390/jcm15031027)
Supplement: Supplementary file 1 [file jcm-15-01027-s001.zip › jcm-4089588-supplementary/S1_Datasets_comparison.pdf]

Supplementary Table S1. Descriptive statistics of the age, anthropometric measurements, BMI, SSNDT, experience and weekly load of training in three independent cohorts of the students. One-way ANOVA test results.

	2023 year; N = 106				2024 year; N = 45				2025 year; N = 33				statistic	
	mean	95%CI		SD	mean	95%CI		SD	mean	95%CI		SD	F	p
Age [y]	20,57	20,38	20,75	1,03	20,37	20,21	20,53	0,77	20,51	20,26	20,76	1,02	1,10	0,328
Body height [cm]	186,27	184,99	187,54	6,98	185,02	183,59	186,45	6,81	186,51	184,84	188,18	6,80	1,20	0,315
Body weight [kg]	80,22	78,70	81,74	8,33	80,39	79,03	81,75	6,48	81,63	79,41	83,85	9,03	0,72	0,488
BMI [kg/m^2]	23,08	22,81	23,34	1,47	23,47	23,22	23,72	1,18	23,42	22,98	23,86	1,78	2,20	0,113
SSNDT [mm]	7,47	7,00	7,95	2,60	7,90	7,32	8,49	2,80	8,26	7,30	9,22	3,90	1,51	0,223
Experience [years]	4,58	4,44	4,73	0,81	4,06	4,00	4,11	0,27	4,09	4,01	4,17	0,34	26.57	0.001
Load [h/week]	8,58	7,90	9,25	3,72	7,09	6,59	7,59	2,39	7,09	6,53	7,65	2,28	8.16	0.001

Bonferrooni:

Experience: 2023 vs 2024 p = <0.001, 2023 vs 2025 p = 0.005, 2024 vs 2025 p = 1.000

Load: 2023 vs 2024 p = <0.001, 2023 vs 2025 p < 0.001, 2024 vs 2025 p = 1.000
