# Supplementary material for: Sit-to-Stand Navicular Drop Test-Based Injury Risk Zones Derived from a U-Shaped Relationship in Male University Athletes
Source: J Clin Med. 2026 Jan 27;15(3):1027. doi: 10.3390/jcm15031027 (PMC12898812; doi:10.3390/jcm15031027)
Supplement: Supplementary file 1 [file jcm-15-01027-s001.zip › jcm-4089588-supplementary/S2_Risk_gradients.pdf]

Supplementary Table S2. Continuous SSNDT risk gradient and structural diagnostics from segmented regression.

Panel A. Local odds ratio per 1-mm increase in SSNDT (continuous risk gradient)

SSNDT (mm)	Log-odds slope β′(x)	Cl lower	Cl upper
3	0,82	0,59	1,15
4	0,91	0,69	1,20
**5.47 (minimum risk zone)**	1,01	0,81	1,25
6	1,11	0,95	1,31
7	1,23	1,09	1,39
8	1,36	1,22	1,52
9	1,51	1,32	1,72
10	1,67	1,39	2,00
11	1,84	1,46	2,33

Panel B. Structural diagnostics from segmented regression

Model	Breakpoints	ψ (mm) (breakpoints)	AIC	BIC
Linear	0	-	243.84	251.07
Segmented	1	5.094 (SE 0.527)	217.72	232.17
Segmented	2	3.85; 10.25	3544.28	3565.96

Notes: Local OR values were derived from the fixed-effect component of the quadratic logistic model and represent the relative change in injury odds associated with a 1-mm increase in SSNDT at each point.
The single breakpoint identified by the segmented model corresponds closely to the minimum-risk region estimated by the quadratic model. The two-breakpoint specification showed numerical instability and extreme penalization, indicating overfitting and lack of interpretability.

Segmented regression was used as a structural diagnostic tool rather than a primary inferential model, and its results were interpreted in conjunction with the mixed-effects quadratic model.
